# Nonenzymatic Glucose Sensor Using Bimetallic Catalysts

**DOI:** 10.1021/acsami.3c10167

**Published:** 2023-12-20

**Authors:** Rashmi Ghosh, Xiao Li, Matthew Z. Yates

**Affiliations:** Department of Chemical Engineering, University of Rochester, Rochester, New York 14627, United States

**Keywords:** glucose sensor, electrochemical, bimetallic catalyst, glucose oxidation, nonenzymatic catalysis

## Abstract

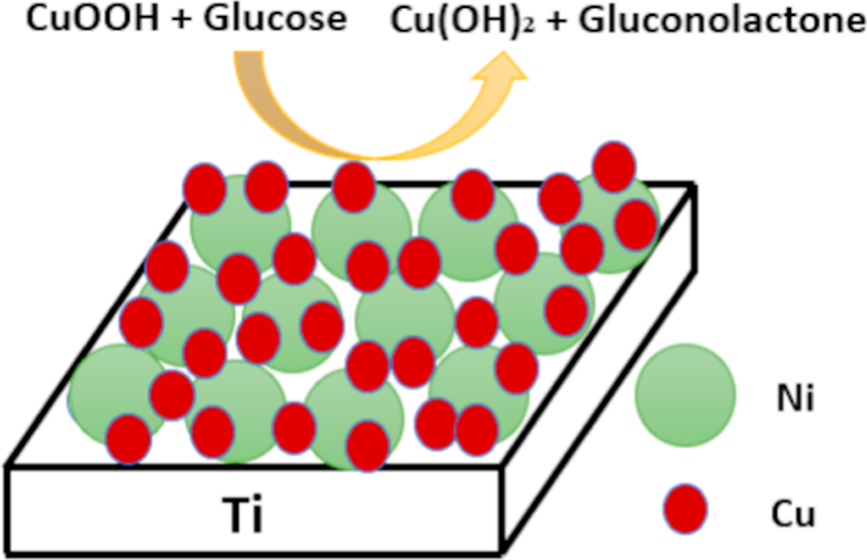

Bimetallic glucose oxidation electrocatalysts were synthesized by two electrochemical reduction reactions carried out in series onto a titanium electrode. Nickel was deposited in the first synthesis stage followed by either silver or copper in the second stage to form Ag@Ni and Cu@Ni bimetallic structures. The chemical composition, crystal structure, and morphology of the resulting metal coating of the titanium electrode were investigated by X-ray diffraction, energy-dispersive X-ray spectroscopy, X-ray photoelectron spectroscopy, and electron microscopy. The electrocatalytic performance of the coated titanium electrodes toward glucose oxidation was probed using cyclic voltammetry and amperometry. It was found that the unique high surface area bimetallic structures have superior electrocatalytic activity compared to nickel alone. The resulting catalyst-coated titanium electrode served as a nonenzymatic glucose sensor with high sensitivity and low limit of detection for glucose. The Cu@Ni catalyst enables accurate measurement of glucose over the concentration range of 0.2–12 mM, which includes the full normal human blood glucose range, with the maximum level extending high enough to encompass warning levels for prediabetic and diabetic conditions. The sensors were also found to perform well in the presence of several chemical compounds found in human blood known to interfere with nonenzymatic sensors.

## 1 Introduction

Fast and reliable detection of glucose is of great scientific and technological importance both in healthcare and in industrial analytical applications. The measuring of glucose concentration is important in the food and biotechnology industries as well as in clinic diagnostics, where monitoring glucose levels plays a critical role in treating diabetic patients. The most common glucose sensors use an electric current response from an electrode covered with a glucose oxidation catalyst. When an appropriate electric potential is applied to the electrode, the catalytic oxidation of glucose results in a measurable current that can be correlated to the glucose concentration. Traditionally, the glucose oxidation catalyst is an immobilized enzyme, but there is growing interest in developing nonenzymatic sensors.^1^ Enzymatic glucose sensors suffer from drawbacks due to denaturization of enzymes, which can occur due to variations in temperature, pH, and humidity.^2−4^ Nonenzymatic sensors have been developed using a wide range of electrocatalysts.^5−11^

The main advantage of nonenzymatic sensors is their robust stability under a range of storage and operating conditions that would destroy enzymes. Depending on how they are constructed, nonenzymatic sensors have the potential to also be of much lower cost than enzymatic sensors. The detection of glucose using nonenzymatic metal-based sensors via direct oxidation has its own set of challenges, however. Since the most important application is in glucose monitoring for diabetes treatment, the nonenzymatic sensors should ideally work on biological samples, such as blood plasma or whole blood. In addition, the sensors should provide accurate measurements for patients whose blood glucose level falls outside the normal range of 4.4–6.6 mM.^12^ Blood sugar concentration for healthy people ranges between 4.0 and 5.4 mM in fasting and up to 7.8 mM postprandial. Prediabetes is when values are between 5.5 and 6.9 mM in fasting and between 7.8 and 11.0 mM postprandial. Blood sugar levels are >7.0 mM in fasting and >11.1 mM postprandial for people suffering from diabetes. Accuracy over a wide range of glucose concentration is required as well as performance in the presence of numerous other biochemical compounds found in blood.

Some of the earliest nonenzymatic sensors used precious metal-based electrocatalysts. Besides being expensive, they have limited sensitivity and selectivity, surface poisoning from adsorbed intermediates, and interference from chloride ions.^7,13^ There is ongoing development of transition metal-based glucose sensors, with particular focus on nickel- and cobalt-based catalysts, to overcome the limitations of precious metal catalysts.^1^ Transition-metal catalysts have been created that have good stability, low detection limit, fast response, high sensitivity, and low cost.^14−17^ The lower selectivity of metal-based sensors with respect to glucose oxidase enzyme-based sensors is found to be offset by engineering the morphology and surface of the electrode. Here, we report on the development of novel bimetallic catalysts synthesized directly on the surface of a titanium sensor electrode. By carrying out short electrochemical reduction reactions, metal nanoparticles can be uniformly deposited on the electrode surface. A second electrochemical reduction reaction can then be carried out that deposits nanoparticles of a different type of metal on top of the first. The bimetallic film that is formed is composed of two types of transition-metal nanoparticles deposited separately and not an alloy. Nickel was chosen for the first layer deposition because it is one of the most widely studied glucose oxidation catalysts.^1,18^ For the second layer, copper and silver were chosen for investigation because of their known performance in catalyzing glucose oxidation alone or when coupled with other metals.^9,18−20^ The fabricated bimetallic thin film electrodes have high surface area and higher catalytic active sites and were found to have excellent electrochemical properties for use in glucose sensing.

## 2 Experimental Procedure

### 2.1 Materials

Tris(hydroxymethyl)aminomethane (>99.8%), AgNO_3_ (>99.0%), NaCl, NaOH, NaNO_3_, NiSO_4_·6H_2_O (>99.0%), CuSO_4_·5H_2_O (>99.0%), NH_4_Cl (99.9%), and dextrose were purchased from Sigma-Aldrich. Ethanol (200 Proof) was obtained from Koptec. Titanium (Ti, 0.89 mm thick) and platinum (Pt, 0.127 mm thick) were obtained from Alfa Aesar. All chemicals were used as received. Deionized water was used throughout the experiments.

### 2.2 Synthesis of Coatings

The Ti plate (8 × 8 × 0.89 mm^3^) was cleaned by ultrasonication using detergent water and ethanol consecutively, followed by rinsing with deionized water. During the two-stage electrochemical deposition of bimetallic thin films, a two-electrode system was used with a Pt plate as the anode and a Ti plate as the cathode. The two electrodes were submerged in the electrolyte solution and maintained at a fixed distance of 10 mm. An aqueous electrolyte solution for the electrodeposition of Ni nanoparticles was prepared by adding 50 mM NaCl, 50 mM tris(hydroxymethyl)aminomethane, 0.75 mM NiSO_4_·6H_2_O, and 18.75 mM NH_4_Cl in 125 mL of water under continuous stirring, with pH adjusted to 7.3 by addition of HCl. The electrolyte solution was heated to 95 °C using an oil bath for 20 min prior deposition. A constant current of 62.5 mA/cm^2^ was passed for a duration of 2, 4, 6, 8, and 12 min to deposit a uniform layer of Ni nanocrystals. After the deposition, the coating was washed with deionized water and dried in atmospheric air. The Ni-coated plate was used as the electrode for a second electrochemical reduction reaction to deposit either silver or Cu nanoparticles.

For the second-stage of Ag deposition on the Ni-coated Ti plate, an aqueous electrolyte solution was prepared by adding 50 mM NaNO_3_, 50 mM tris(hydroxymethyl)aminomethane, 0.5 mM AgNO_3_, and 12.5 mM NH_4_Cl in 125 mL of water. The Ni-coated Ti plate and the Pt plate were used as the cathode and anode, respectively. The reaction was carried out at a constant current density of 62.5 mA/cm^2^ at room temperature with constant stirring for 4 min. For the second-stage of Cu deposition on a Ni-coated Ti plate, an electrolyte solution was prepared by adding 50 mM NaCl, 50 mM tris(hydroxymethyl)aminomethane, 0.5 mM CuSO_4_, and 12.5 mM NH_4_Cl in 125 mL of water. A constant current of 62.5 mA/cm^2^ was passed at room temperature under constant stirring for 4 min to deposit Cu nanoparticles on top of Ni crystals. At the end of the electrodeposition process, the composite coatings were thoroughly rinsed with deionized water and dried at room temperature. Ag and Cu were found to form as separate nanoparticles rather than an alloy with Ni. The bimetallic films were named Ag@Ni and Cu@Ni, respectively.

### 2.3 Scanning Electron Microscopy

The surface composition and morphology of the coating were obtained using a Zeiss-Leo DSM982 scanning electron microscope. The microscope was equipped with a Phoenix energy dispersive X-ray photoelectron spectroscope, which was used in analyzing the composition of the constituent ions on the surface of the coatings.

### 2.4 X-ray Diffraction

The crystal structure of the coating was studied by using powder X-ray diffraction (XRD). The powdered coating samples were obtained by removing the nanoparticles from six identical samples prepared under the same conditions using ultrasonication in water followed by low-temperature heating to remove the water. The XRD data from the resulting powder samples were from a Philips model PW3020 diffractometer with Cu Kα radiation (λ = 1.5418 Å) measured in the range of 10–80°.

### 2.5 X-ray Photoelectron Spectroscopy

The surface composition of the coating was studied by using a Kratos AXIS Ultra DLD X-ray photoelectron spectrometer fitted with a monochromatic Al anode X-ray gun (Kα = 1486.6 eV) and a spectrum electron analyzer. The survey spectra were collected using a pass energy of 160 eV whereas high-resolution spectra were collected using a pass energy of 20 eV. All the spectra were fitted using CasaXPS software. For the X-ray power source, a mono Al filament was used with an emission current of 10 mA and anode HT at 15 kV. The C peak appeared at 285.00 eV at the survey spectra and was used as the internal reference for charging correction.

### 2.6 Inductively Coupled Plasma Mass Spectroscopy

Inductively coupled plasma mass spectroscopy (ICP-MS) was used to measure the concentration of Ni, Ag, and Cu ions that leached into the solution during the electrocatalytic detection of glucose molecules using the fabricated electrodes. The ICP-MS system used was a PerkinElmer Model NexION 2000 in STD and KED modes with 4.2 mL/min He flow. The reference material tested was Seronorm blood with accepted values of 980 ± 80, 9.2 ± 1.9, and 9.7 ± 0.4 ppb and measured values being 991.4, 8.62, and 9.35 ppb for Cu, Ni, and Ag measurement, respectively. The Ni-coated Ti plate, Ni–Ag-coated Ti plate, and Ni–Cu-coated Ti plate were placed in 0.1 M NaOH solution containing 1 mM glucose at room temperature for 25 cycles of cyclic voltammetry (CV) measurements from −0.6 to 1.1 V. The resulting glucose solutions in NaOH collected after CV experiments were sent for ICP-MS analysis. All the measurements were done in triplicate, and the mean was reported. Additionally, a control experiment was carried out using a Ti plate without a coating for comparison.

### 2.7 Electrochemical Glucose Oxidation

A conventional three-electrode system consisting of a platinum plate as the counter electrode, Ti plate coated with metal nanoparticles as the working electrode, and a Ag/AgCl reference electrode was used to characterize the electrochemical properties of the fabricated electrodes. The electrochemical analysis was studied using a homemade potentiostat/galvanostat.^21^ CV experiments were conducted between −0.6 and 0.7 V for studying the electrocatalytic activities of the electrode materials and their electrokinetic properties in the presence or absence of glucose in 0.1 M NaOH. Electrochemical experiments were conducted at constant potential condition with successive addition of glucose stock solution to record the amperometric responses of the samples to an increasing glucose concentration. The calibration curves plotted by using the amperometric data were then used to calculate the limit of detection, sensitivity, and linear range of response for glucose for all three sample electrodes. The current density mentioned in the calibration curves was calculated using the geometric surface area and not the electrochemically active surface area (ECSA). Selectivity was similarly tested by successively adding glucose along with other potential interferants in a 0.1 M NaOH solution at constant potential condition. Reproducibility was tested by measuring the peak current in the presence of 1 mM glucose for each type of sample. Stability of the investigated electrode materials was tested by conducting 25 CV cycles in 0.1 M NaOH in the presence of glucose molecules followed by doing ICP-MS measurements of the electrolyte solution to determine if any metal ions leached out into the solution during the extended CV runs.

## 3 Results and Discussion

### 3.1 Morphology and Composition of Coatings

**Figure 1:**
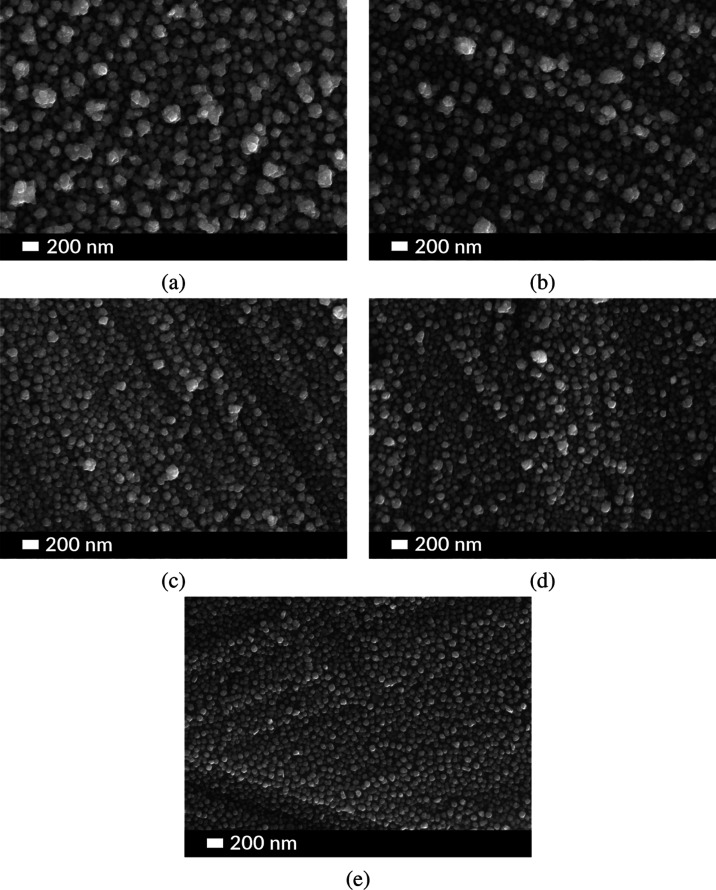
SEM images of the Ni-coated Ti plate using a constant current of 62.5 mA/cm^2^ at 95 °C for a duration of (a) 12, (b) 8, (c) 6, (d) 4, and (e) 2 min.

The morphology and size of the nanoparticles in the single transition metal-based film or bimetallic composite film were studied in detail using scanning electron microscopy (SEM). The three samples, namely, Ni, Cu@Ni, and Ag@Ni, were synthesized using the previously described electrolytic deposition process at a constant current density. In all the experiments, a 25 mm × 25 mm sized platinum plate was used as the anode and an 8 mm × 8 mm sized titanium plate was used as the cathode. All of the electrochemical deposition reactions were carried out in a two-electrode system. Crystalline coatings of Ni with nanosphere-shaped particles were fabricated at varying reaction times, including, 2, 4, 6, 8, and 12 min at a reaction temperature of 95 °C under constant current density of 62.5 mA/cm^2^. As seen in Figure 1, the size and distribution of the Ni nanoparticles varied with the reaction time. At the highest reaction time of 12 min, larger Ni crystals were formed sometimes overlapping each other with an increasing tendency of agglomeration at irregular spots on the surface of the coatings. At the lowest reaction time of 2 min, smaller distinct Ni crystals were formed which were spherical in the shape. The electrochemically deposited Ni crystals on the Ti plate acted as the cathode for a second stage deposition of Ag or Cu.

Coatings of Ni nanocrystals deposited for shorter reaction times had void regions without Ni at some places on the surface of the electrode which reduced the effective surface area and catalytic activity toward glucose molecules. At higher reaction times, electrodeposited Ni nanocrystals tended to agglomerate with materials being formed in the bulk, which reduced the effective outer surface area. Hence, for all of the second-stage deposition reactions, Ni coatings electrochemically deposited for a reaction time of 8 min on a Ti plate were chosen as the working electrode. The Ni coating deposited using the 8 min reaction time from here on will be termed sample Ni. Sample Ni was used as a cathode in the second-stage deposition of Cu or Ag crystals. The average diameter of Ni crystals in both the single metal and bimetallic samples was approximately 75 nm as measured from the SEM images.

**Figure 2:**
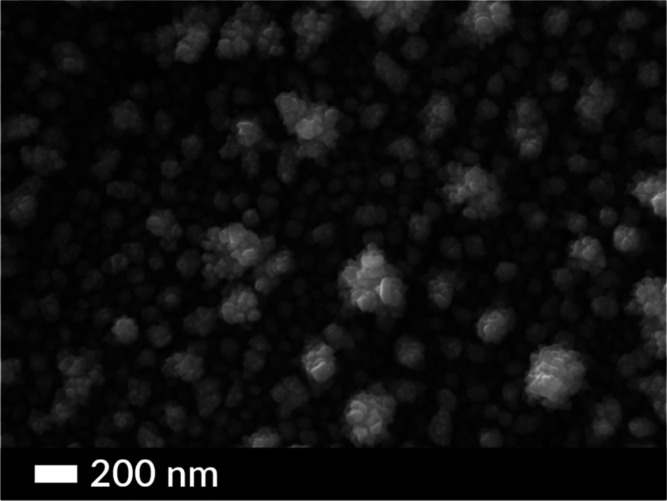
SEM image of two-stage electrodeposition of the Ni-coated Ti plate using a constant current of 62.5 mA/cm^2^ at 95 °C for 8 min (first stage) and Cu on the Ni-coated Ti plate using a constant current of 62.5 mA/cm^2^ at room temperature for 4 min (second stage).

**Figure 3:**
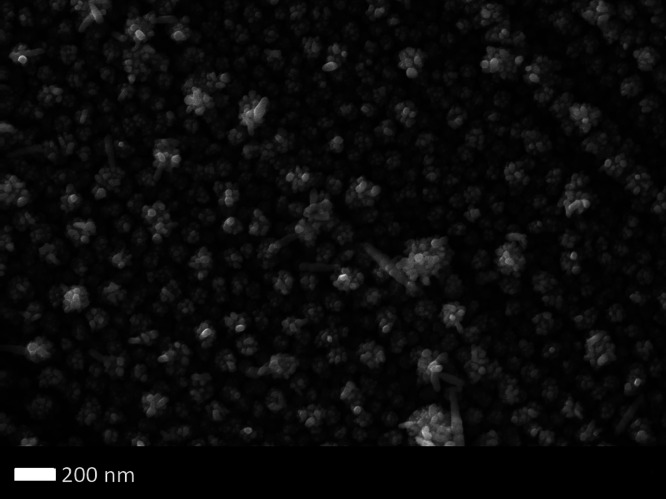
SEM image of two-stage electrodeposition of the Ni-coated Ti plate using a constant current of 62.5 mA/cm^2^ at 95 °C for 8 min (first stage) and Ag on the Ni-coated Ti plate using a constant current of 62.5 mA/cm^2^ at room temperature for 4 min (second stage).

For the sample Cu@Ni as shown in Figure 2, copper (Cu) nanoparticles electrodeposited on Ni crystals showed a tendency to agglomerate at the surface of Ni. At higher copper concentration, these agglomerates formed a single nanostructure, whereas at an optimized low Cu concentration distinct Cu nanocrystals were observed on the surface. The diameter of an individual Cu nanoparticle averaged 30 nm, whereas the size of these agglomerated Cu@Ni nanostructures varied from 100 to 250 nm. For the sample Ag@Ni as shown in Figure 3, Ag nanoparticles were deposited uniformly and distinctly all over the Ni crystals. The diameter of the Ag nanoparticles averaged 20 nm. The compositions of the individual as well as composite metallic coatings were studied using electron dispersive X-ray spectroscopy (EDX). Elemental composition was measured at three different positions of the sample, and the data were averaged. The Ni concentration was 20 wt % averaged across all the samples, whereas the amount of Ag and Cu was found to be approximately 5 and 4 wt %, respectively. The average elemental composition along with standard deviation in measurement is shown in Table 1. Additionally based on the individual elemental ion mapping images (the Supporting Information file), the transition-metal ions were found to be uniformly distributed on the surface of the Ti substrate.

**Table 1:** Elemental Composition of the Catalytic Coatings

sample	Ni (wt %)	Ag (wt %)	Cu (wt %)
Ni	18.59 ± 1.50	nil	nil
Ag@Ni	14.80 ± 3.49	4.78 ± 1.69	nil
Cu@Ni	23.31 ± 0.84	nil	3.56 ± 0.46

**Figure 4:**
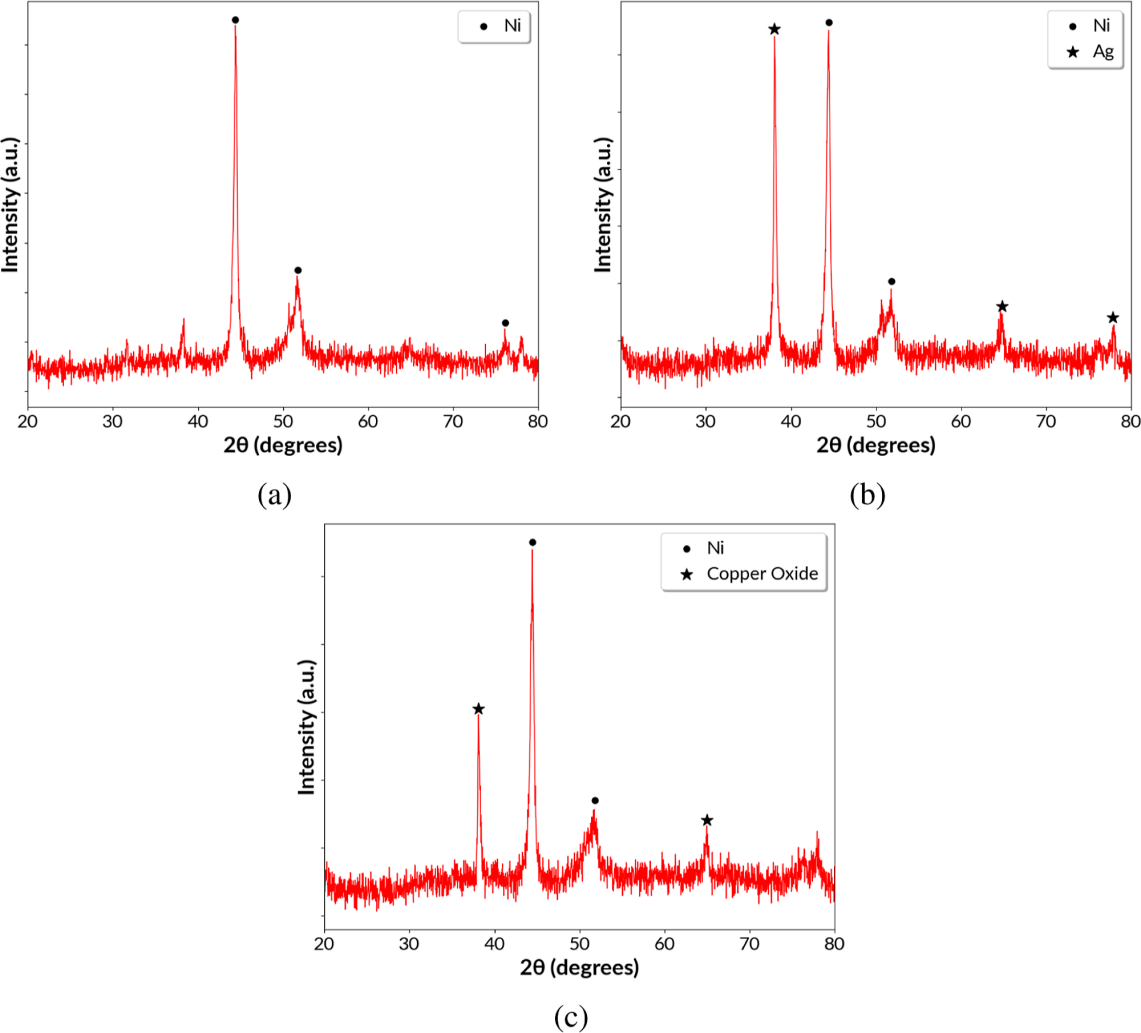
XRD spectra of (a) Ni coating, (b) Ag@Ni coating, and (c) Cu@Ni coating.

Six samples each of Ni, Cu@Ni, and Ag@Ni were synthesized under the same conditions used for the samples analyzed in Table 1 and then removed from the Ti surface using an ultrasonic bath for XRD analysis. Dry metallic powder was collected by briefly heating the ultrasonicated solution containing metal nanoparticles at 80 °C. Ultrasonication was used to effectively remove the coatings from the Ti substrate; however, the possibility of breaking the nanoparticles in further small fragments remained. As the nanoparticles were heated for drying, oxide peaks were recorded in the XRD spectra. The XRD spectra of all three samples are shown in Figure 4. The diffraction patterns of Ni, Ag, CuO, and Cu matched the standard reference peaks with ICDD card numbers of 03-065-0380, 01-077-6577, 41-0254, and 01-071-4607, respectively. The crystallite size of the metal nanoparticles may be calculated using the Debye–Scherrer relation
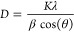
1where *D* is the mean size of the crystalline domain, *K* is the dimensionless shape factor, λ is the X-ray wavelength, β is the line broadening at half the maximum intensity [full width at half-maximum(fwhm)], and θ is the Bragg angle.

For the sample Ni as shown in Figure 4a, strong Ni diffraction peaks were seen at 44.40, 51.74, and 76.15°. These three peaks represented Ni crystalline planes (111), (200), and (220), respectively. Calculating the Ni crystallite size using the Debye–Scherrer relation for the (111) peak gave a result of 28.8 nm which was much smaller than the nanoparticle size obtained from SEM images (75 nm). The difference in size is possibly due to nanoparticles being polycrystalline. For the sample Cu@Ni, the Ni peaks were seen at 44.46 and 51.80° as shown in Figure 4c representing the crystalline planes (111) and (200), respectively. Strong diffraction peaks were also recorded at 38.10 and 64.95° due to the CuO crystalline planes of (111) and (022), respectively. Observation of CuO peaks instead of Cu is due to the heating of the samples at 80 °C. The (111) crystalline plane of Cu is located at 43.379° which is located near the (111) plane of Ni and the two peaks are probably convoluted, leading to the broadening of fwhm (β) for the peak located at 44.46°. Hence, the XRD spectrum peaks were studied as a qualitative measurement and not as a quantitative measurement of the crystallite size. Nanoparticle sizes were successfully measured from SEM images. In Figure 4b for the sample Ag@Ni, the Ni peaks were observed at 44.42 and 51.81° representing the crystalline planes (111) and (200), respectively. Strong diffraction peaks were also recorded at 38.06, 64.82, and 77.85° representing the crystalline planes (111), (220), and (311), respectively, for the Ag nanoparticles. The (200) crystalline plane of Ag is located at 44.599° which is near to the (111) plane of Ni. Here also, there was a possibility that the two peaks were convoluted, leading to the broadening of fwhm (β) for the peak located at 44.42°.

The chemical states of the metal atoms near the surface were investigated in detail using X-ray photoelectron spectroscopy (XPS) (the Supporting Information file). The results confirmed the presence of metallic Ni in all three samples. Metallic copper was detected in the Cu@Ni sample, along with copper oxide. Metallic Ag was detected in the Ag@Ni sample. The XPS data confirm the XRD results.

### 3.2 Glucose Oxidation: Electrocatalytic and Electrokinetic Activity

**Figure 5:**
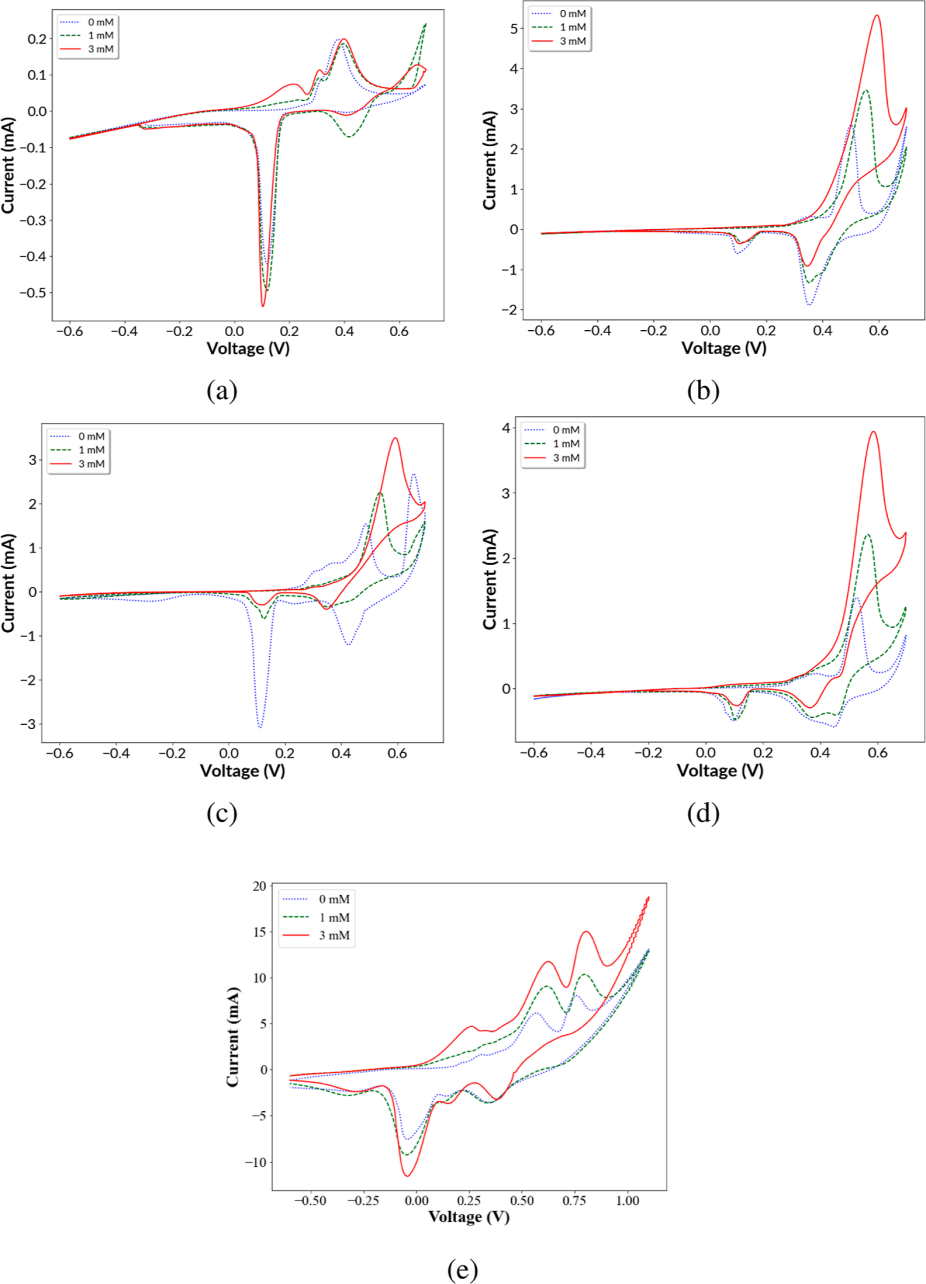
CV curves of (a) bare Ti control, (b) Ni, (c) Ag@Ni, (d) Cu@Ni, and (e) Ag@Ni coatings in 0.1 M NaOH with 0, 1, and 3 mM of glucose. Scans (a–d) are from −0.6 to 0.7 V at a rate of 10 mV/s. Scan (e) is from −0.6 to 1.1 V at a rate of 50 mV/s in order to show both Ag oxidation peaks.

The electrocatalytic activity of bare Ti, Ni, Cu@Ni, and Ag@Ni toward glucose oxidation was examined by CV in a 0.1 M NaOH aqueous solution at a scan rate of 10 mV/s. CV experiments were conducted between −0.6 and 0.7 V using glucose concentrations of 0, 1, and 3 mM. An additional CV was conducted for Ag@Ni between −0.6 and 1.1 V (Figure 5e) using glucose concentrations of 0, 1, and 3 mM to allow the completion of the anodic scan of the Ag@Ni electrocatalyst. As shown in Figure 5a, bare Ti showed a small oxidation peak that was independent of the glucose concentration. Bare Ti was inert and acted as a control for all the reactions. All the three other samples showed oxidation peaks that increase with the glucose concentration, indicating catalytic activity due to glucose oxidation.

The transition metal-based nonenzymatic glucose sensor depends on the transition metal/oxide surface getting activated in the presence of hydroxide ions in a basic environment to act as a catalyst for glucose oxidation. The three transition metals concerned in our study are nickel (Ni), copper (Cu), and silver (Ag), forming glucose sensor materials, namely, Ni, Cu@Ni, and Ag@Ni. Under the alkaline conditions of our experiment, metallic Ni in the presence of hydroxide ions is expected to get transformed to Ni(OH)_2_ which further reacts with hydroxide ions to form nickel oxyhydroxide (NiOOH) at 0.54 V during the anodic scan.^18,22^ This NiOOH intermediate generated acts as an electrocatalyst for the oxidation of glucose to gluconolactone and then itself gets reduced back to Ni(OH)_2_ during the cathodic scan.^23−25^
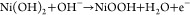
2
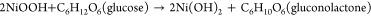
3

In regards to the catalyst Cu@Ni, copper (Cu) at atmospheric conditions gets oxidized to copper oxide (CuO).^26^ Besides Ni transforming to Ni(OH)_2_, CuO reacts with the water to form Cu(OH)_2_ which then further changes to CuOOH forming a combined electrocatalyst of CuOOH/NiOOH. Glucose gets oxidized to gluconolactone in the presence of CuOOH/NiOOH as the electrocatalyst.^27,28^
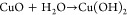
4
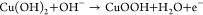
5
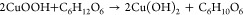
6

In regard to the electrocatalyst Ag@Ni, glucose is oxidized in two steps in the presence of Ag-based electrocatalysts. As shown in Figure 5e, during the anodic scan Ag nanoparticles in Ag@Ni (1 mM glucose curve) form Ag_2_O/AgOH in the presence of hydroxide ions during the first peak at 0.62 V and then Ag_2_O/AgOH changes to AgO during the second anodic peak at 0.80 V.^29,30^ Similarly, Ni(OH)_2_ forms the intermediate NiOOH. The AgO/NiOOH intermediate then acts as an electrocatalyst for the oxidation of glucose to gluconolactone which is further oxidized to gluconic acid. During the cathodic scan at peak locations of 0.34 and −0.05 V, Ag_2_O was regenerated from Ag and then Ag was again regenerated from Ag_2_O.^31−34^ The peak identifications are consistent with the mechanism of silver nanoparticle-catalyzed glucose oxidation described by Poletti Papi et al.^29^
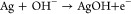
7
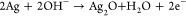
8
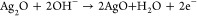
9
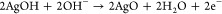
10
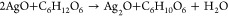
11
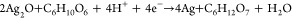
12

In the presence of glucose, all three catalysts Ni, Cu@Ni, and Ag@Ni exhibited higher anodic redox peak current (*I*_pa_) and the observed redox anodic peak potential (*E*_pa_) resembled their respective redox units of Ni^3+^, Ni^3+^/Cu^3+^, and Ni^3+^/Ag^2+^, validating the effective participation of the active catalytic centers of the electroredox couples toward oxidation of glucose. The increase in the anodic current due to an increase of glucose concentrations from 0 to 3 mM establishes the effective electrocatalytic activity of all the three catalysts Ni, Cu@Ni, and Ag@Ni.^18,24,35^

The high effective surface area of all the samples with 75, 30, and 20 nm sized nanocrystals (based on SEM images) was a major driving force behind the excellent electrocatalytic activity toward glucose oxidation. In this study, we used geometric area of the electrode for calculating the performance of the electrodes toward glucose oxidation. In future work, we measure the ECSA. Since, the electrodes were made of thin films of nanostructured transition metals, the ECSA is likely higher than the geometric surface area. In the case of bimetallic thin film deposition, due to the second set of nanoparticles deposited on the surface of Ni nanocrystals, the effective surface area is expected to increase and enhance the number of active catalytic sites. In a later discussion, it will be shown that the overall electrocatalytic performance of bimetallic catalysts exceeds that of Ni alone.

**Figure 6:**
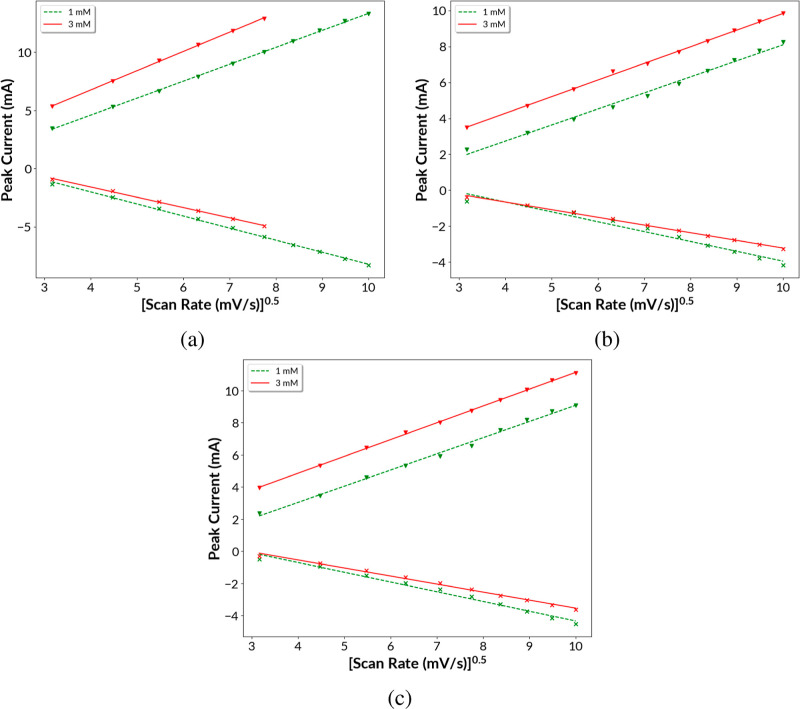
Peak current vs square root of the scan rate for the CV curves (between −0.6 and 0.7 V) at different scan rates (10–100 mV/s) for (a) Ni, (b) Ag@Ni, and (c) Cu@Ni with 1 and 3 mM glucose in 0.1 M NaOH solution. The anodic peak is positive, and cathodic peak is negative. Data are truncated for 3 mM glucose with the Ni sample because the anodic peak was cut off at the four highest scan rates.

To understand the electrokinetics of the catalytic glucose oxidation system for all the three samples Ni, Cu@Ni, and Ag@Ni, a set of CV measurements were recorded with the scanning speed ranging from 10 to 100 mV/s in increments of 10 mV/s in the presence of 1 and 3 mM glucose (the Supporting Information). As expected, the redox peak current density increased with an increase in the scan rate. In addition, the peak-to-peak separation between oxidation and reduction peaks increased with the scan rate, and the ratio of oxidation/reduction real current was not unity. The CV results indicate that the reaction is partially irreversible or quasi-reversible.^36,37^ The CV data at various scan rates were converted to a Randles–Sevcik plot of peak current versus the square root of the scan rate as shown in Figure 6 for each of the three catalytic electrodes. For a surface electrochemical reaction that is diffusion controlled, it is expected to follow the Randles–Sevcik equation
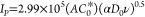
13where, *D*_0_ is the diffusion coefficient of the analyte molecules, *A* is the area of the electrode, *C*_0_^*^ is the concentration of the analyte molecules that diffuses, α is the transfer coefficient, and ν is the potential sweep speed.^38^ The excellent linear fit of the data in Figure 6 is strong evidence that the electrocatalytic process is controlled by the diffusion of glucose molecules into the electrode/electrolyte interface.^39−42^

For all three catalysts Ni, Cu@Ni, and Ag@Ni with an increasing glucose concentration from 0, 1, to 3 mM, the anodic peak current was also found to increase. For our diffusion-controlled process, as more glucose molecules are present in the solution in a high glucose concentration, higher number of glucose molecules diffuses to the electrocatalyst surface requiring a higher number of redox units of Ni^3+^, Ni^3+^/Cu^3+^, and Ni^3+^/Ag^2+^ to act as electrocatalysts for glucose oxidation. From this increase in the anodic peak current with an increasing glucose concentration, we can conclude a successful formation of the redox units, like, Ni^3+^ from Ni^2+^ and participation of Ni^3+^ in the catalytic oxidation of glucose.^43,44^

### 3.3 Glucose Oxidation: Amperometric Measurement

**Figure 7:**
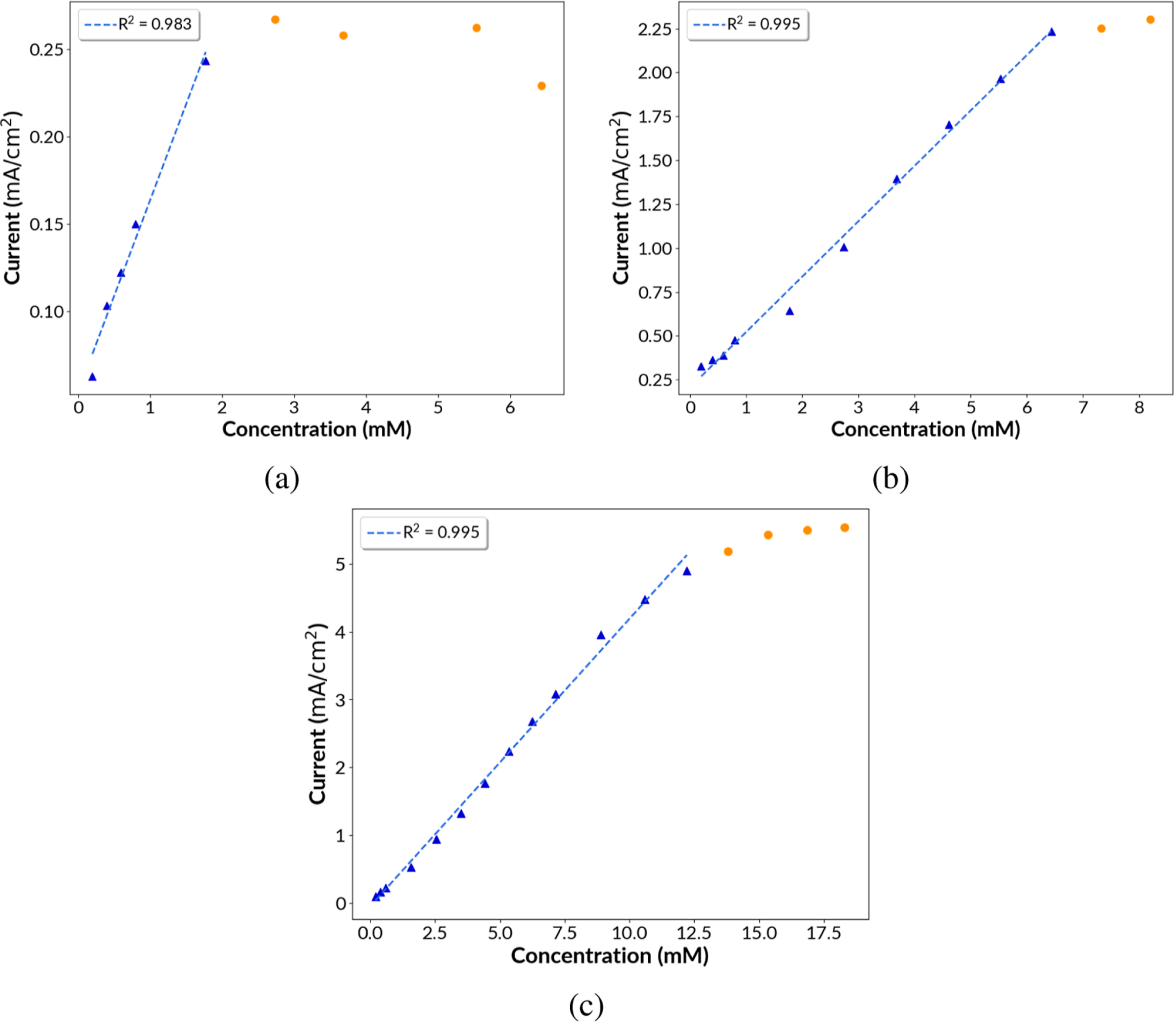
Amperometric calibration plots for (a) Ni, (b) Ag@Ni, and (c) Cu@Ni.

For glucose concentration measurement, amperometry is typically used where the electrode is held at a fixed potential, and current response is correlated to the glucose concentration. This requires amperometric calibration of the electrodes against solutions of a known glucose concentration. A series of amperometric measurements were taken to calibrate each of the three types of electrodes (the Supporting Information). The applied fixed potential was +0.54, +0.62, and +0.62 V vs Ag/AgCl for Ni, Cu@Ni, and Ag@Ni. Before the addition of glucose to the NaOH solution to conduct amperometry experiments, the electrodes were stabilized in 0.1 M NaOH solution at the fixed activating potential for 10 min. A quasi-stationary current of 28.52 μA/cm^2^ (±0.51 μA/cm^2^), 79.77 μA/cm^2^ (±8.75 μA/cm^2^), and 275.76 μA/cm^2^ (±14.92 μA/cm^2^) was recorded for Ni, Cu@Ni, and Ag@Ni, respectively, at the end of the stabilization period at a zero glucose concentration. The quasi-stationary current was determined by averaging the current response in the last 30 s of the stabilization period. After stabilization and blank current measurement, the current response was measured in the same 60 mL of 0.1 M NaOH as a 120 mM glucose stock solution was added incrementally. After each addition of glucose, the solution was stirred for 40 s, followed by a 260 s stabilization period. The current response to a particular glucose concentration was obtained by averaging the current readings in the last 30 s of the stabilization period. Figure 7 shows the resulting calibration curve for each of the three types of electrodes with the glucose concentration in mM as the *x* axis and the current response in mA/cm^2^ (based on geometric surface area of the electrode) as the *y* axis.

The calibration curves in Figure 7 were used to calculate the limit of detection, sensitivity, and linear range of response. The linear response range was defined as the bracketed glucose concentration range for which a linear regression could accurately represent the glucose concentration versus the current response. Sensitivity was calculated as the slope of the linear regression. The detection limit was calculated using the formula: (3 × sd)/*S* (where, sd is the standard deviation of the blank signal and *S* is the slope of the calibration curve). Table 2 compares the figures of merit for all three samples Ni, Cu@Ni, and Ag@Ni. The samples Ni, Ag@Ni, and Cu@Ni exhibited a linear range of 0.2–1.8, 0.2–6.4, and 0.2–12.2 mM, respectively, with a high coefficient of determination of 0.983, 0.995, and 0.995, respectively. Cu@Ni showed the highest sensitivity of 420 μA/(mM cm^2^) and the lowest for Ni with a value of 110 μA/(mM cm^2^). The detection limits for Ni, Cu@Ni, and Ag@Ni were 14, 62.5, and 140 μM, respectively. The linear range of response as well as the sensitivity improved significantly for the bimetallic samples Cu@Ni and Ag@Ni in comparison to Ni. The sensitivity of Ag@Ni and Cu@Ni was 3× and 4× higher than that of Ni due to higher active catalytic sites in the bimetallic samples. The linear range of response for Ni was 0.2–1.8 mM of the glucose concentration. The upper limit of the linear response range increased from 1.8 to 6.4 and 12.2 mM for Ag@Ni and Cu@Ni, respectively. The linear response of the Cu@Ni electrode spans the entire normal glucose concentration range in human blood, and the upper limit of 12.2 mM is high enough to encompass warning levels indicating prediabetic and diabetic conditions.

**Table 2:** Glucose Sensing Performance

sample	linear range (mM)	sensitivity 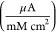	detection limit (μM)
Ni	0.2–1.8	110	14.0
Ag@Ni	0.2–6.4	320	139.9
Cu@Ni	0.2–12.2	420	62.5

### 3.4 Comparison to Other Nonenzymatic Sensors

The literature review on nonenzymatic electrochemical glucose sensors by Hwang et al. shows that noble metal, nonprecious transition metal/metal oxides, and metal alloy/composite catalytic materials have been designed and developed for electrochemical glucose sensing.^15^ Noble metal-based electrodes show a high linear range of response toward glucose oxidation covering the human blood sugar levels of 2–8 mM; however, they showed poor sensitivity and were easily poisoned by interfering molecules besides being expensive. The nonprecious transition-metal counterpart had good sensitivity as well as anti-interference properties. However, the majority of the transition metal-based glucose sensing electrodes have a linear range of response that does not sufficiently cover human blood glucose concentrations. Furthermore, extremely high sensitivity in the case of some of the transition metal/metal oxide electrodes was due to high surface area substrates in the form of foam or porous substrates.^15^ In this work, a broad linear response of 0.2–12.2 mM was achieved with a sensitivity of 420 μA/(mM cm^2^) for the sample Cu@Ni. In one of the research articles, Anu Prathap et al. prepared CuO nanoparticles in the presence of tartaric acid/citric acid/amino acid and achieved a linear range of response 0.9–16.0 mM for glucose oxidation. However, during the amperometric experiment, the author modified a Pt electrode with the prepared CuO. There was no discussion if the underlying Pt electrode played a role in the high linear range recorded. Additionally, the author reported a sensitivity of only 9.02 μA/mM.^45^ Similarly, in another work, Subramanian et al. deposited rGO/Ni(OH)_2_ composites on Au electrodes to get a linear range of response of 15 μM–30 mM with a sensitivity of 11.4 mA mM^–1^ cm^–2^. However, here also there was no discussion if the underlying gold substrate played a role in the high value of figure of merits.^46^ In another work by Zhang et al., the author started with a Cu–Zr–Ag ingot and developed metallic glass ribbons by melt spinning followed by dealloying and other procedures, like anodizing, to finally form nanowires on nanoporous substrates. No detailed discussion was carried out about the dimension of the nanoporous substrate and how that might impact the result of a linear range of glucose detection of up to 15 mM with a sensitivity of 1310 μA/(mM cm^2^).^47^ A glucose oxidase-based enzymatic glucose sensor, Gox/Au–ZnO/GCE, prepared by Fang et al. recorded a linear range of response of 1–20 mM with a sensitivity of 1.409 μA/mM.^48^ Here also, in addition to enzyme, gold was used in the electrode. Jeong et al. prepared another enzymatic sensor, Gox/3D MoS_2_/graphene aerogel, with a linear range of glucose detection of 2–20 mM and a sensitivity of 3.36 μA/mM.^49^ In a recent review, Sehit and Altintas tabulated the performance of an enzyme-based glucose sensor with a widest linear range of glucose detection reported as 0–25 mM and a highest value of sensitivity noted as 289 μA/(mM cm^2^) for a different electrode material.^50^ Additionally, a very recent review of copper-based glucose sensors reported figures of merits from the literature reports of 520+ sensors.^51^ From this list, we find only eight studies that reported a higher upper limit of linear response range to the glucose concentration while maintaining a greater sensitivity than our best catalyst (Cu@Ni; linear response range: 0.2–12.2 mM and sensitivity: 420 μA/(mM cm^2^). Our work also has the added advantage of a simple low-cost preparation of bimetallic catalysts and sensors. There were no expensive materials involved in the catalyst synthesis, and the resulting catalyst-coated solid titanium plate is used directly as the sensing electrode without requiring other materials to facilitate electrode transfer during the glucose oxidation reaction.

### 3.5 Glucose Oxidation: Selectivity, Stability, and Reproducibility

**Figure 8:**
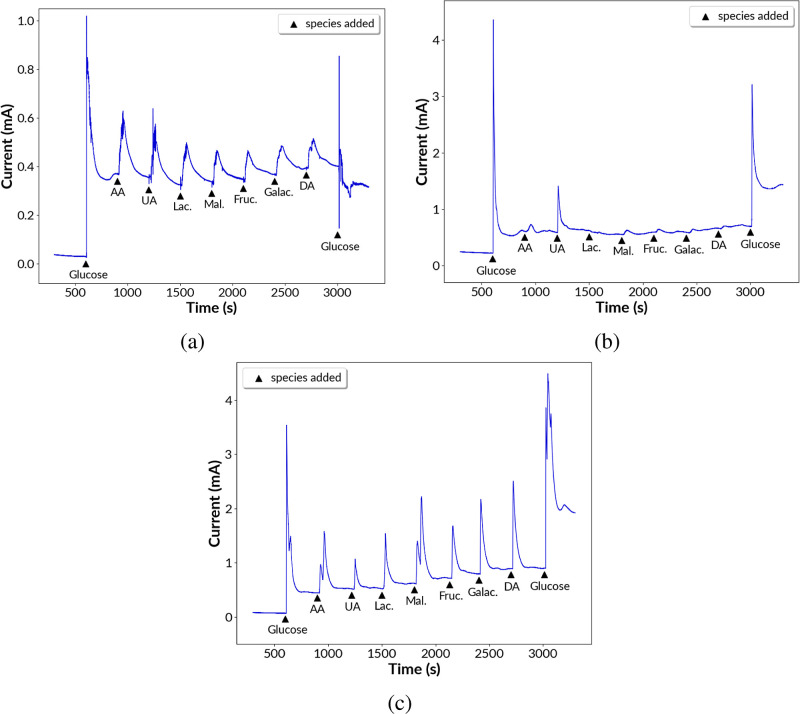
Selectivity toward glucose oxidation in the presence of ascorbic acid (AA), uric acid (UA), lactose (Lac.), maltose (Mal.), fructose (Fruc.), galactose (Galac.), and dopamine (DA) due to the samples of (a) Ni coating, (b) Ag@Ni coating, and (c) Cu@Ni coating.

One of the important parameters to be considered for fabricating a sensor material for the catalytic oxidation of glucose is its ability to eliminate the interfering responses generated by the species with similar electroactivity as that of the target analyte. In this work, for the Ni, Cu@Ni, and Ag@Ni samples, the selectivity of glucose was tested in the presence of the common interferents, including ascorbic acid (AA), uric acid (UA), dopamine (DA), lactose, maltose, fructose, and galactose. The experiments were carried out at fixed applied potentials of +0.54, +0.62, and +0.62 V for Ni, Cu@Ni, and Ag@Ni, respectively. The changes in the current response after the addition of the glucose and interferent solutions were studied. After each addition of glucose or an interferent, the solution was stirred for 40 s followed by 260 s of the stabilization period. The current response to a particular chemical addition was obtained by averaging the current response readings in the last 30 s of the stabilization period. After the initial stabilization of 10 min, 1 mM glucose was added to 60 mL of 0.1 M NaOH followed by 0.2 mM of each of the interferents every 300 s, and at the end, another 2 mM glucose was added. From Figure 8a, it was seen that for Ni, the current response after the addition of 1 mM glucose was 0.37 mA and the current increased to only 0.40 mA after addition of all the seven interferents with each constituent’s concentration being 0.2 mM. After the addition 2 mM glucose at the end there was no significant increase in the current response for the sample Ni. The electrode material sample Ni surface was positioned and deactivated in the presence of all the interfering chemicals and did not respond to the glucose molecules added at the end of the reaction. In the case of Cu@Ni, (Figure 8c) the current was stabilized to 0.45 mA after addition of the initial 1 mM glucose and the current increased at an average rate of 10% after the addition of each interferent. Further work on improving the selectivity of Cu@Ni needs to be pursued. Here, the Cu@Ni material electrode was fully functional even after adding all the interferents, and the current increased to 1.93 mA after the addition of 2 mM glucose at the end. The selectivity toward catalytic glucose oxidation for the sample Ag@Ni was recorded as shown in Figure 8b. The initial current after 1 mM of glucose addition was measured as 0.62 mA and the average current increment after the addition of each of the interferents was only 2%. Ag@Ni was fully stable and functional until the end of the selectivity test and recorded an increased current response of 1.44 mA at the end after the addition of 2 mM glucose. The selectivity performance of Ag@Ni was attributed to the fine uniform deposition of Ag nanoparticles on the surface of Ni nanoparticles and to the individual material properties of Ni and Ag.

The reproducibility of each of the electrode samples was tested by fabricating four replicates of each of Ni, Cu@Ni, and Ag@Ni and then measuring the anodic oxidation current at +0.54, +0.62, and +0.62 V, respectively, from the oxidation of 1 mM of glucose in 60 mL of 0.1 M NaOH solution. The mean anodic oxidation current was calculated, along with the standard deviation. The relative standard deviations for Ni, Cu@Ni, and Ag@Ni were 9.67, 6.47, and 5.12%, respectively.

The stability of Ni nanospheres electrodeposited on the Ti surface as well as the stability of bimetallic nanocrystalline Cu@Ni and Ag@Ni after repeated CV experiments in the presence of 1 mM glucose in 0.1 M NaOH was studied using ICP-MS analysis. The ICP-MS measurements were conducted for the Ni, Cu, and Ag ions that leached out into NaOH solution after the repeated 25 CV cycles at 50 mV/s between −0.6 and 1.1 V. Three replicates of each of the samples were tested along with a control experiment where CV was done with bare Ti without any material deposited on its surface. Low amount of metal ions leached into the solution as shown in the Table 3 validating the stability of the materials deposited on the surface of Ti even after long experimental runs.

**Table 3:** Concentration of Metal Ions in the Electrolyte Solution after the Glucose Oxidation Reaction

sample	Ni conc. (ppb)	Ag conc. (ppb)	Cu conc. (ppb)
control	3.55 ± 0.10	1.15 ± 0.36	13.34 ± 1.78
Ni	3.58 ± 0.30	1.68 ± 1.09	15.02 ± 0.16
Ag@Ni	2.95 ± 0.13	17.44 ± 4.51	15.73 ± 1.04
Cu@Ni	3.46 ± 0.08	1.49 ± 0.81	41.91 ± 4.45

## 4 Conclusions

Electrochemical deposition, being a clean, easy-to-operate, and low-cost method, was effectively used to fabricate nanocrystals of nickel as well as composite nanostructures of nickel–copper and nickel–silver. A multistage electrochemical deposition technique was optimized to deposit 20 nm sized Ag nanoparticles uniformly on 75 nm sized Ni crystals. This method of two-stage electrodeposition was replicated for other metals, thereby depositing 30 nm sized Cu nanostructures on the Ni nanospheres. The materials prepared in this work were tested for their catalytic activity, sensitivity, selectivity, and linear range of response toward glucose oxidation. On all of the figures of merit, Cu@Ni and Ag@Ni excelled in comparison to the nanocrystalline Ni alone. The combined participation of the bimetallic material promoted higher catalytic surface area and better electron transportation leading to a wide linear range of amperometric response as well as high sensitivity. Cu@Ni recorded a wide 0.2–12.2 mM linear range of glucose concentration detection with the best sensitivity of 420 μA/(mM cm^2^) among all the three electrode materials. The linear range of response for glucose detection for both Cu@Ni and Ag@Ni covered the expected glucose concentration found in a normal human blood sample. Ag@Ni showed the best selectivity toward glucose oxidation in the presence of interferents with only an average 2% increase in current response after each addition of each interferent. All three variants were reproducible and were stable on the surface of titanium after extended reactions. The performance of the transition-metal composites Ag@Ni and Cu@Ni developed in this work was better than the existing literature on the transition metal-based nonenzymatic glucose sensor in terms of a wide linear range of response along with good sensitivity achieved without the presence of any precious metals in the electrode.

## Supporting Information Available

XPS spectra, TEM images, EDX analysis, CV curves, and raw amperometry data used for characterizing sensor performance (PDF)
