# Supplementary material for: Nonenzymatic Glucose Sensor Using Bimetallic Catalysts
Source: ACS Appl Mater Interfaces. 2023 Dec 20;16(1):17–29. doi: 10.1021/acsami.3c10167 (PMC10788829; doi:10.1021/acsami.3c10167)
Supplement: Supplementary file 1 — am3c10167_si_001.pdf [file am3c10167_si_001.pdf]

Supporting Information: Non-Enzymatic Glucose Sensor using Bimetallic Catalyst

Rashmi Ghosh, Xiao Li, and Matthew Z. Yates*

Department of Chemical Engineering, University of Rochester, Rochester, NY 14627, USA

E-mail: myates@che.rochester.edu

Phone: +1-585-273-2335

S1 XPS Measurements

The chemical states of the metal and metal composites were studied using XPS. According to Figure S1(a) in the region scan of Ni 2p for the sample Ni, the core level binding energy peak of Ni $2p_{3/2}$ and Ni Ni $2p_{1/2}$ were recorded at 855.28 eV and 873.13 eV, respectively. Both the core peaks were followed by their satellite peaks at 860.61 eV and 879.19 eV, respectively indicating the formation of metallic Ni. For the sample Ag@Ni, a region scan of Ag in Figure S2(b) showed major peaks of Ag $3d_{5/2}$ and Ag $3d_{3/2}$ at 369.14 eV and 375.16 eV, respectively with a binding energy difference of 6.02 eV in between which confirmed the presence of metallic Ag. Here also, Ni $2p_{3/2}$ and Ni $2p_{3/2}$ peaks were recorded at 856.23 eV and 873.61 eV, respectively along with their satellite peaks confirming the successful formation metallic Ni nanoparticles beneath Ag nanoparticles. In the sample Cu@Ni, major peaks of Cu $2p_{3/2}$ and Cu $2p_{1/2}$ were noted at 932.75 eV and 952.65 eV in Figure S3(b), respectively which confirm the presence of metallic Cu. Further, decoupling of the major peaks lead to the formation of shoulders of Cu $2p_{3/2}$ at 934.72 eV and of Cu $2p_{1/2}$ at 954.71 eV along with the formation of following satellite peaks at 944.42 eV and 963.25 eV indicating the probable formation of an oxide layer due to ambient air. In this sample core level binding energy peak of Ni $2p_{3/2}$ and Ni $2p_{1/2}$ were recorded at 855.75 eV and 873.14 eV followed by their satellite peaks due to the presence of metallic Ni below the Cu nanocrystals.

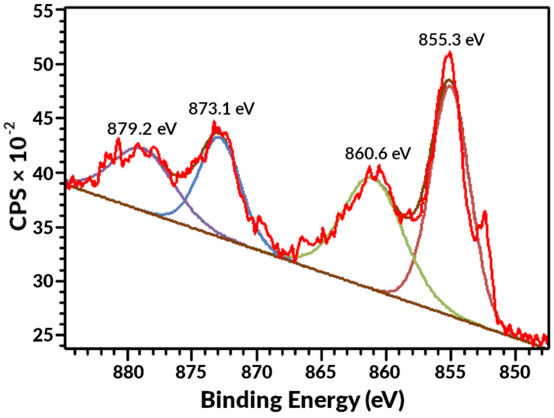

(a)

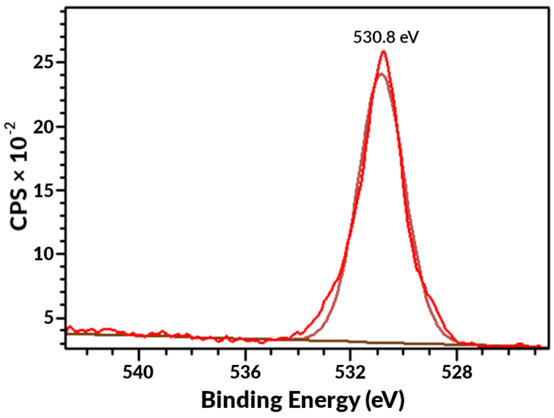

(b)

Figure S1: XPS spectra of (a) Ni (b) O 1s of Ni-coating

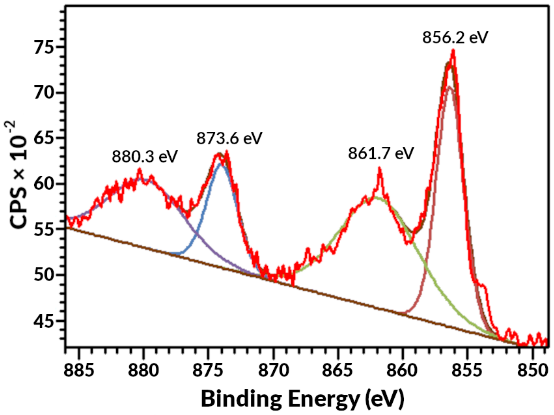

(a)

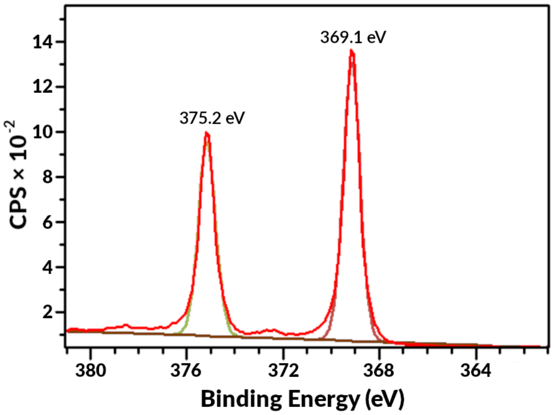

(b)

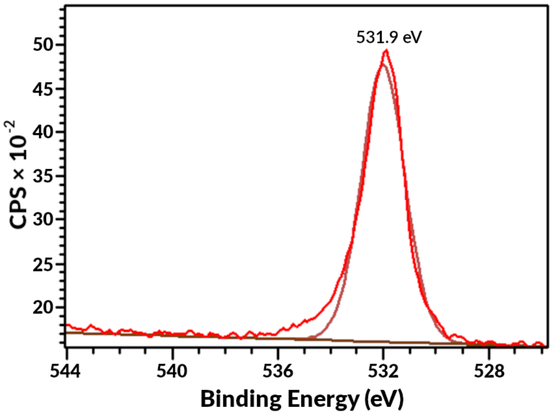

(c)

Figure S2: XPS spectra of (a) Ni (b) Ag (c) O 1s of Ag@Ni-coating.

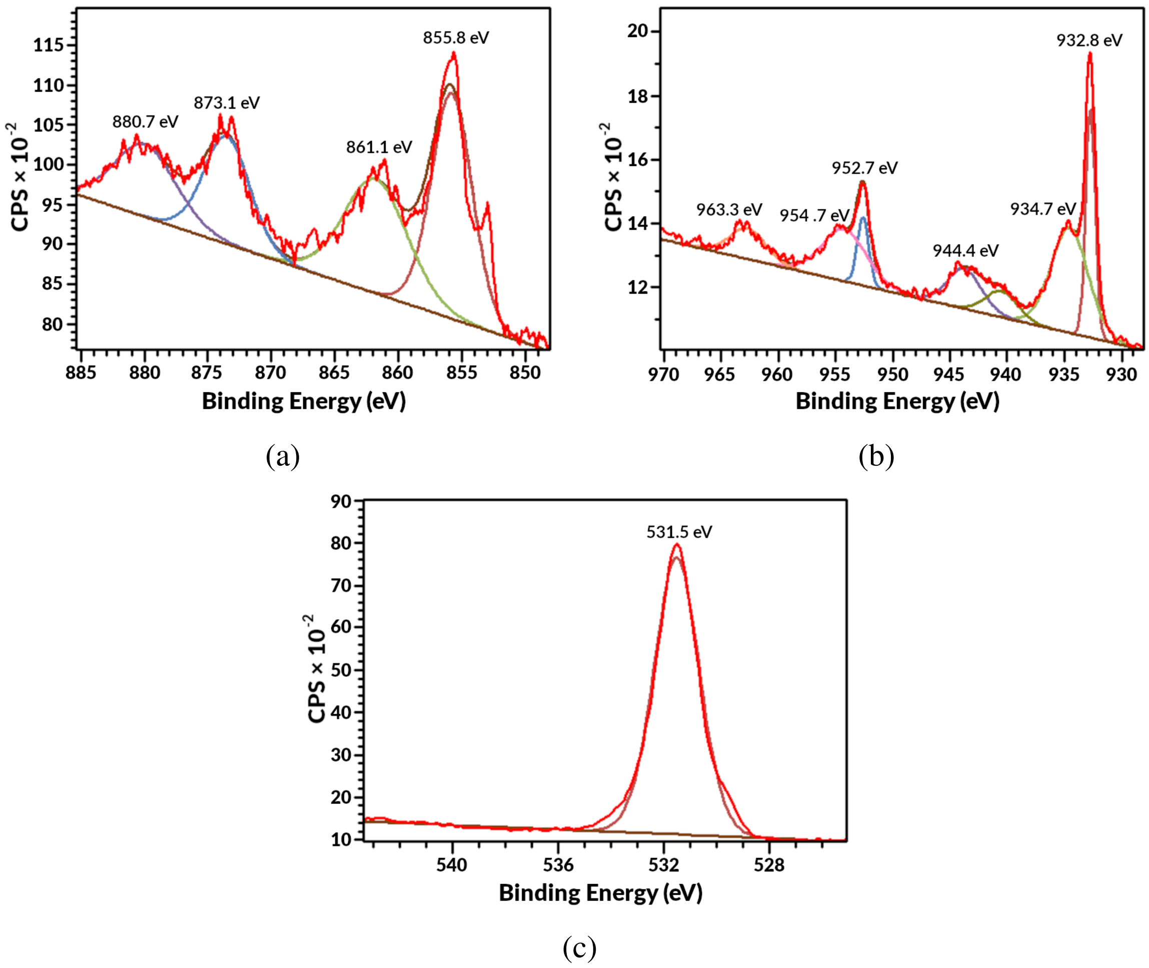

Figure S3: XPS spectra of (a) Ni (b) Cu (c) O 1s of Cu@Ni-coating.

S2 Electron Dispersive X-ray Spectroscopy (EDX)

The elemental composition of the Ni, Ag@Ni, and Cu@Ni coatings was studied using EDX. The average elemental composition for each of the coatings was mentioned in the article. EDX spectra for the coatings Ni, Ag@Ni, and Cu@Ni were recorded in Figures S4(a), S5(a), and S6(a), respectively. In every coating, the individual elements were seen to be uniformly deposited on the Ti substrate from the ion mapping images. The Ti signal was observed due to the underlying substrate.

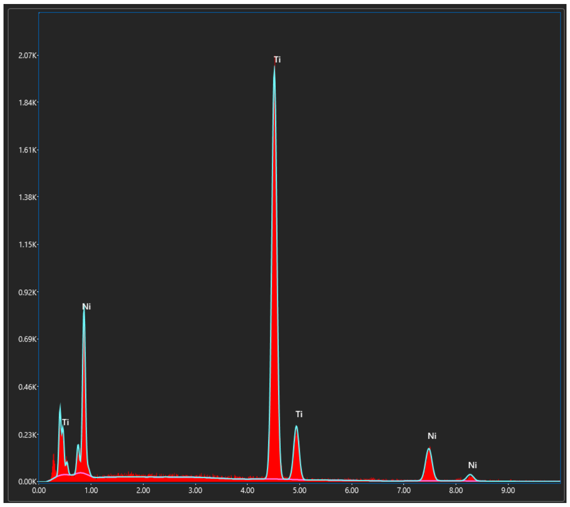

(a)

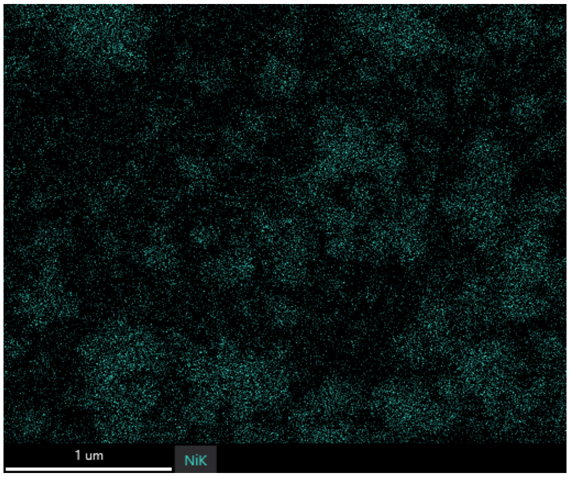

(b)

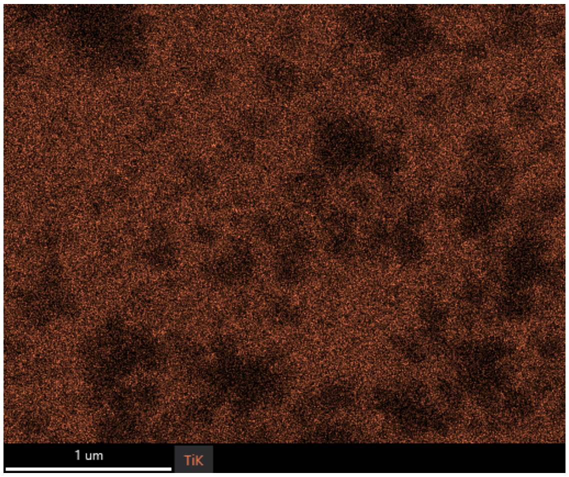

(c)

Figure S4: EDX spectrum of (a) Ni and EDX ion mapping of (b) Ni (c) Ti of Ni-coating

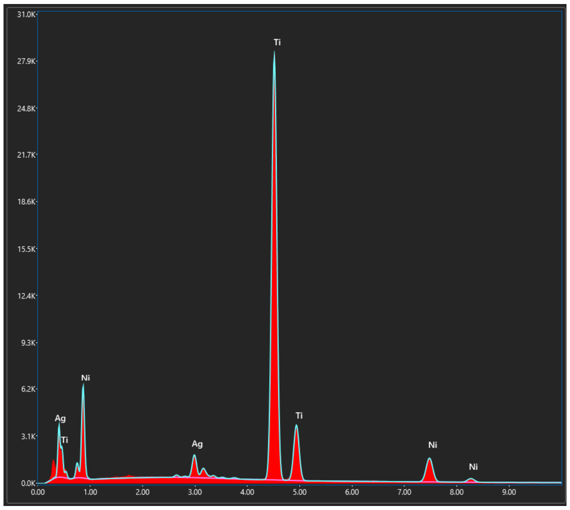

(a)

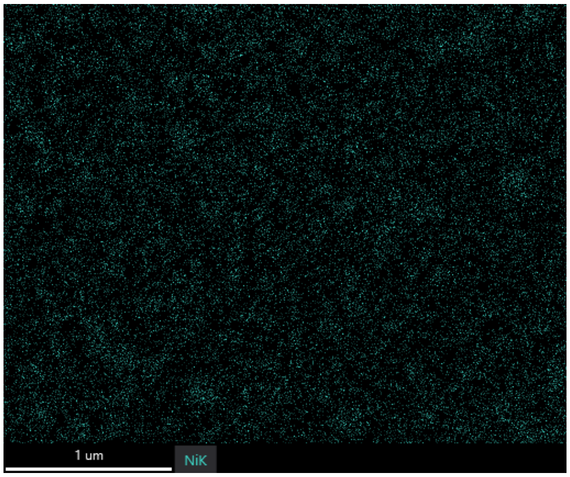

(b)

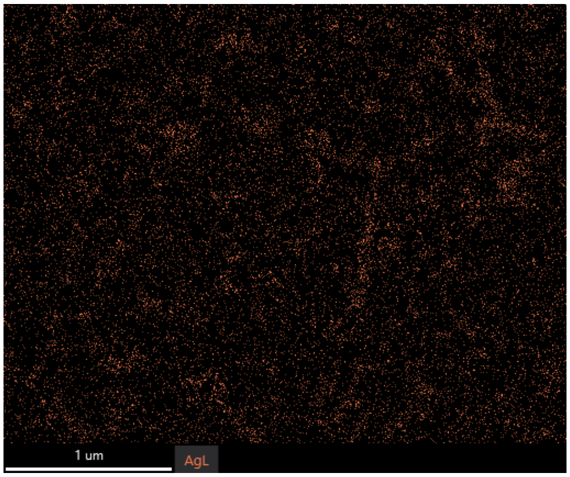

(c)

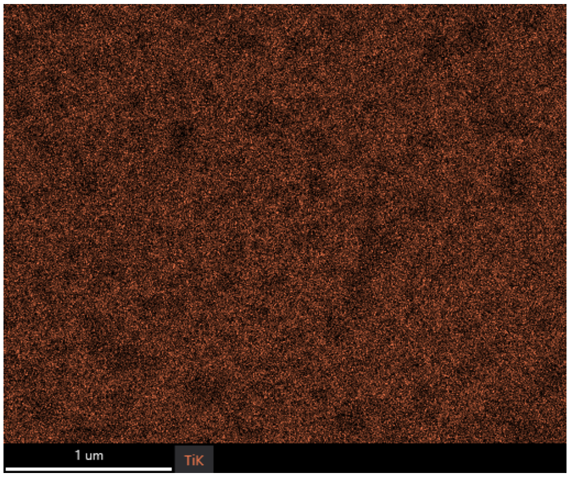

(d)

Figure S5: EDX spectrum of (a) Ag@Ni and EDX ion mapping (b) Ni (c) Ag (d) Ti of Ag@Ni-coating

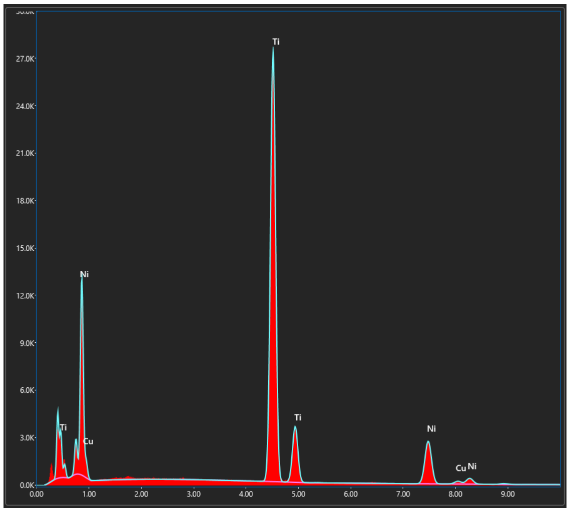

(a)

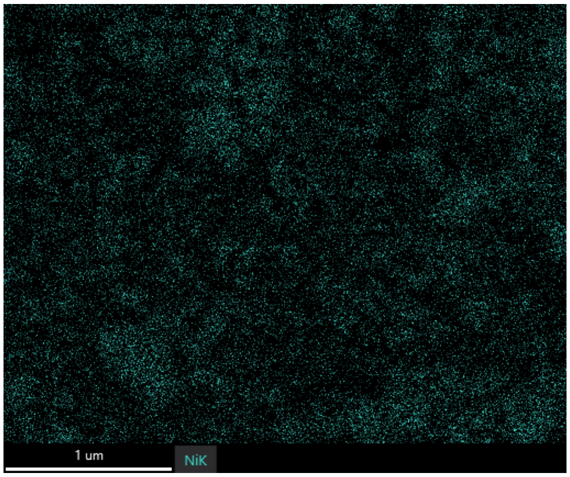

(b)

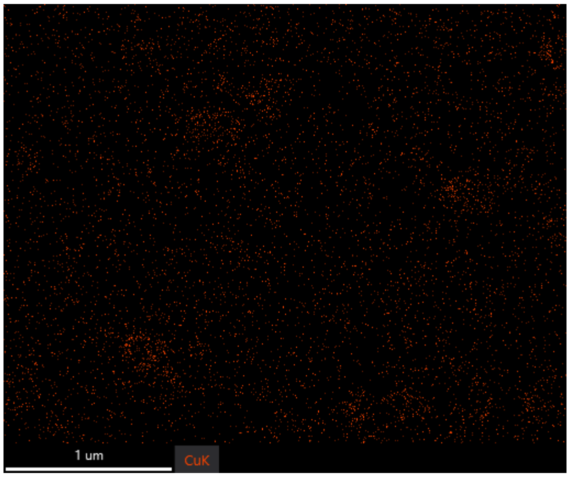

(c)

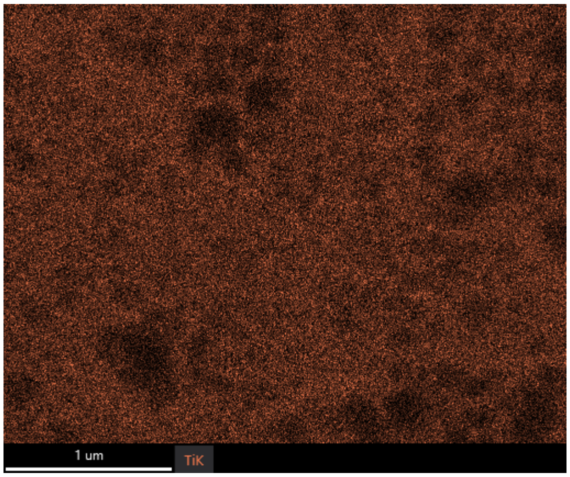

(d)

Figure S6: EDX spectrum of (a) Cu@Ni and EDX ion mapping of (b) Ni (c) Cu (d) Ti of Cu@Ni-coating

S3 Transmission Electron Microscopy (TEM)

The electrocatalysts were directly deposited on the Ti substrate using the cathodic electrochemical reduction process. The TEM analysis could not be directly performed on coatings deposited on the Ti substrate. The electrocatalyst coatings were first removed from the substrate into an isopropanol solution using ultrasonication. The TEM samples were then prepared by evaporative deposition of the isopropanol suspension on the TEM grid. This sample preparation process for the TEM measurement might impact the nanoparticle sizes. From Figures S7(a) and S7(b) of Ni coating, the sizes of agglomerates were measured at 75-150 nm. Additionally, for coatings Ag@Ni (Figures S7(c) and S7(d)) and Cu@Ni (Figures S7(e) and S7(f)), the agglomerates sizes were measured as 20-50 nm and 150-250 nm, respectively.

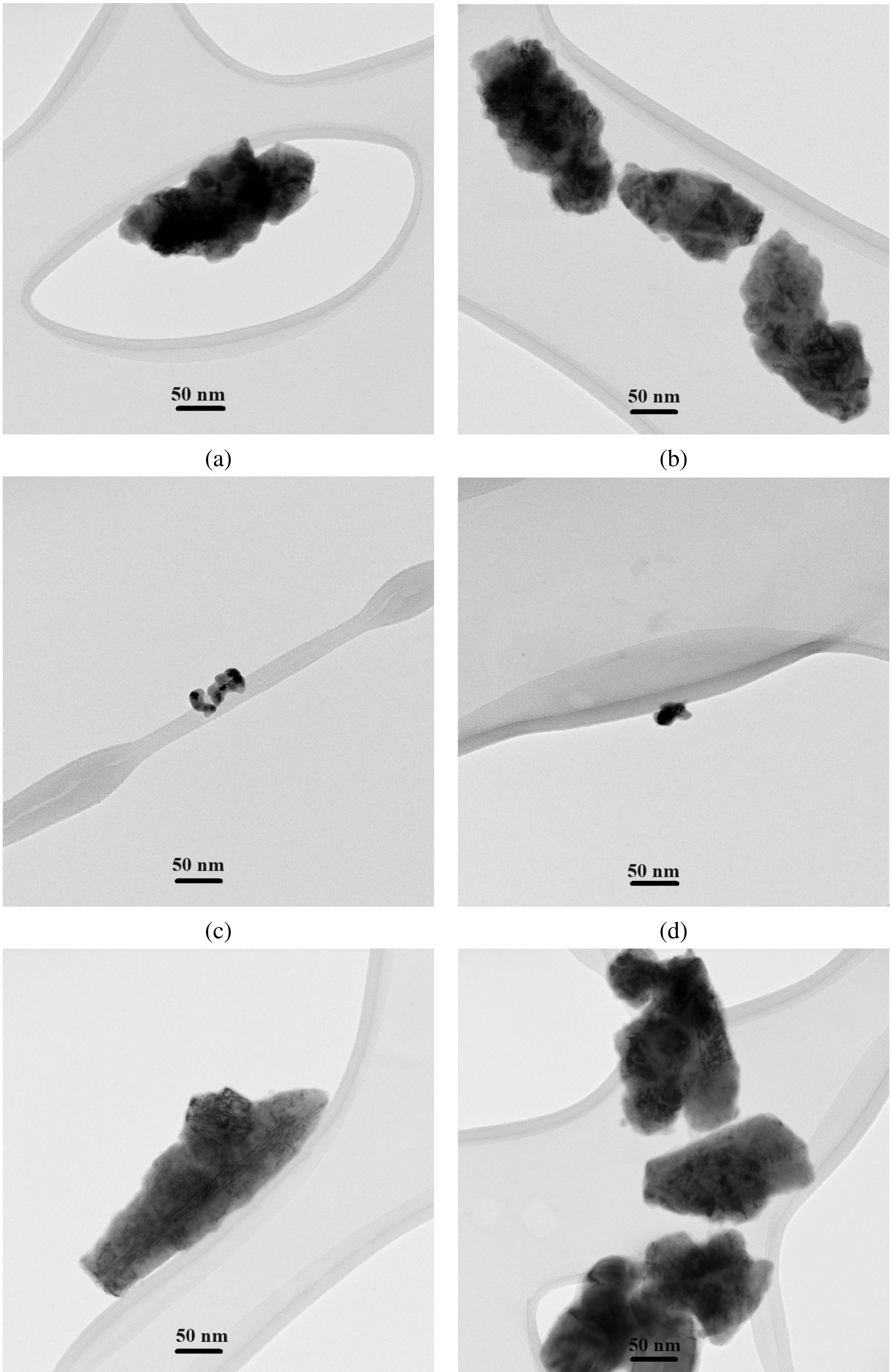

(a) (b) (c) (d) (e) (f)

Figure S7: TEM images of (a), (b) Ni; (c), (d) Ag@Ni; and (e), (f) Cu@Ni

S4 Electrokinetic Activity

A set of cyclic voltammetry (CV) measurements were recorded for the three samples Ni, Ag@Ni, and Cu@Ni to measure their electrokinetic activity in the presence of 1 mM glucose and 3 mM glucose in a 0.1 M NaOH solution. The CV were run between -0.6 V and 0.7 V using a three-electrode system. The scan rate gradually increased from 10 mV/s to 100 mV/s at an increment of 10 mV/s. As discussed in the article, the redox peak current increased with increasing scan rate. The difference between the anodic peak potential and cathodic peak potential increased with increasing scan rate. The ratio of anodic peak current and cathodic peak current was more than 1 signifying that the reactions were quasi-reversible. Tables with the anodic and cathodic peak currents, anodic and cathodic peak potentials, and the peak current ratio were listed for all the samples across the entire scanning speed window for better comparison in this supplemental file. Here, we have included the raw CV data with current (mA) vs voltage (V) plots at varying scan rates for all three samples, Ni, Ag@Ni, and Cu@Ni, in Figures S8(a), S8(c), and S8(e), respectively in presence of 1 mM glucose and in Figures S8(b), S8(d), and S8(f), respectively in presence of 3 mM glucose. Linear relationships were found between peak currents and square root of scan rates for all three samples depicting that the electrocatalytic process was controlled by the diffusion of glucose molecules at the electrode/electrolyte interface (Data and discussion in the main article).

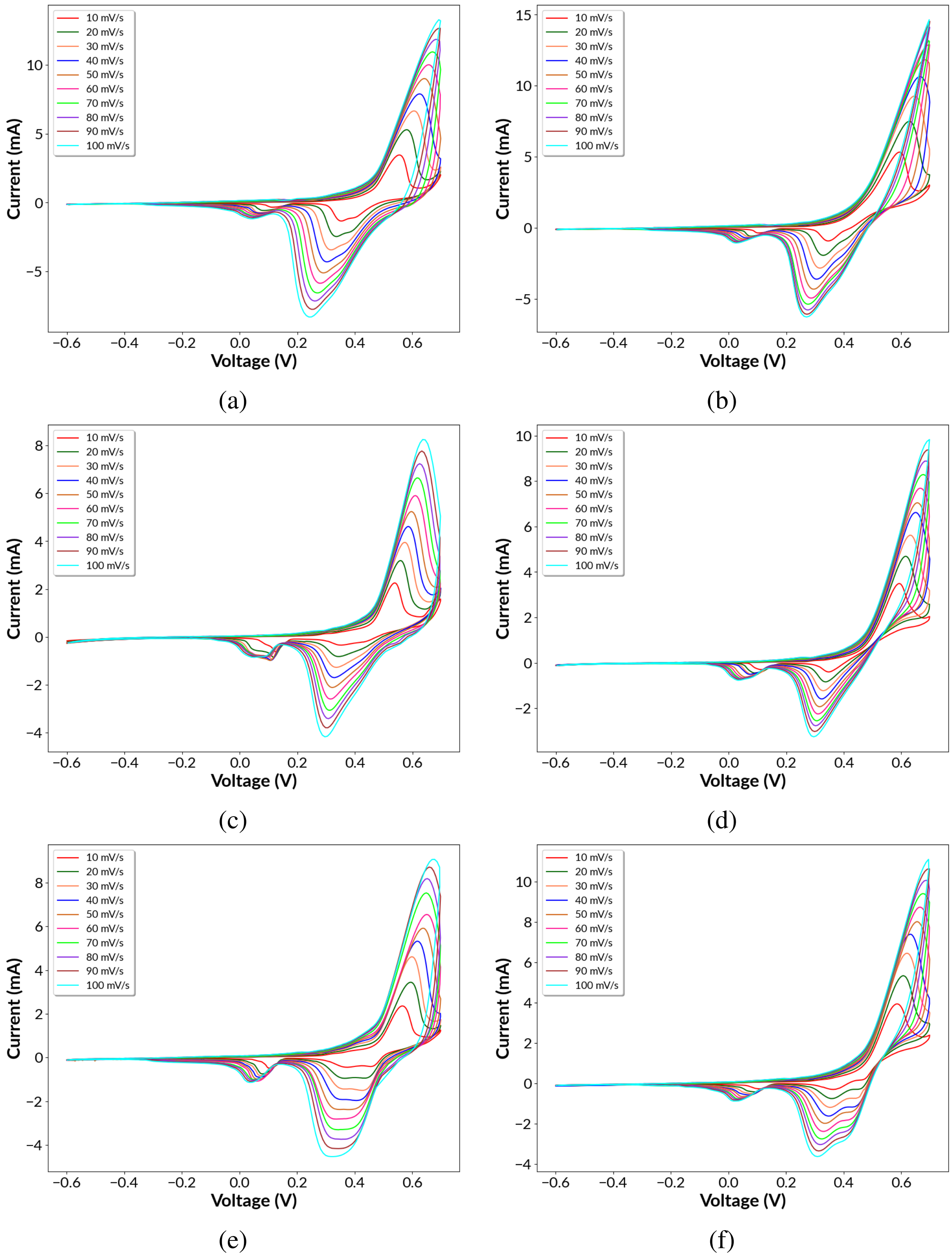

Figure S8: Cyclic voltammetry (CV) at different scan rates (10 mV/s – 100 mV/s) for the (a) Ni in 1 mM glucose, (b) Ni in 3 mM glucose, (c) Ag@Ni in 1 mM glucose, (d) Ag@Ni in 3 mM glucose, (e) Cu@Ni in 1 mM glucose, and (f) Cu@Ni electrodes in 3 mM glucose added to 0.1 M NaOH solution.

Table S1: Electrokinetic data for sample Ni.[a] in 1 mM glucose

ν (mV/s)	$\nu^{1/2}$	E_{pa} (V)	E_{pc} (V)	i_{pa} (mA)	i_{pc} (mA)	ΔE (V)	$\lvert i_{pa}/i_{pc}\rvert$
10	3.16	0.56	0.35	3.46	-1.33	0.20	2.59
20	4.47	0.58	0.34	5.30	-2.46	0.25	2.15
30	5.48	0.61	0.32	6.66	-3.43	0.29	1.94
40	6.32	0.62	0.30	7.90	-4.30	0.32	1.84
50	7.07	0.64	0.29	9.01	-5.10	0.35	1.77
60	7.75	0.65	0.28	10.01	-5.86	0.38	1.71
70	8.37	0.67	0.27	10.95	-6.55	0.40	1.67
80	8.94	0.68	0.26	11.86	-7.14	0.42	1.66
90	9.49	0.70	0.25	12.67	-7.75	0.45	1.63
100	10.00	0.69	0.24	13.28	-8.31	0.45	1.60

Table S2: Electrokinetic data for sample Ni.[a] in 3 mM glucose

ν (mV/s)	$\nu^{1/2}$	E_{pa} (V)	E_{pc} (V)	i_{pa} (mA)	i_{pc} (mA)	ΔE (V)	$\lvert i_{pa}/i_{pc}\rvert$
10	3.16	0.59	0.35	5.33	-0.92	0.25	5.78
20	4.47	0.60	0.33	6.80	-1.93	0.27	3.51
30	5.48	0.60	0.32	7.78	-2.83	0.28	2.75
40	6.32	0.60	0.30	8.17	-3.61	0.29	2.26
50	7.07	0.60	0.30	8.50	-4.31	0.30	1.97
60	7.75	0.60	0.29	8.56	-4.94	0.31	1.73

[a] The symbol ν is scan rate. E_{pa} and E_{pc} are the anodic and cathodic peak potential. i_{pa} and i_{pc} are the anodic and cathodic peak current. ΔE is the potential difference between anodic and cathodic peaks. $|i_{pa}/i_{pc}|$ is the absolute value of the anodic/cathodic peak current ratio.

[a] The symbol ν is scan rate. E_{pa} and E_{pc} are the anodic and cathodic peak potential. i_{pa} and i_{pc} are the anodic and cathodic peak current. ΔE is the potential difference between anodic and cathodic peaks. $|i_{pa}/i_{pc}|$ is the absolute value of the anodic/cathodic peak current ratio.

Table S3: Electrokinetic data for sample Cu@Ni.[a] in 1 mM glucose

ν (mV/s)	$\nu^{1/2}$	E_{pa} (V)	E_{pc} (V)	i_{pa} (mA)	i_{pc} (mA)	ΔE (V)	$\lvert i_{pa}/i_{pc} \rvert$
10	3.16	0.57	0.38	2.36	-0.44	0.19	5.34
20	4.47	0.59	0.36	3.44	-0.95	0.23	3.64
30	5.48	0.60	0.42	4.61	-1.50	0.17	3.07
40	6.32	0.62	0.41	5.32	-1.96	0.21	2.71
50	7.07	0.64	0.38	5.92	-2.38	0.26	2.49
60	7.75	0.65	0.34	6.54	-2.81	0.31	2.33
70	8.37	0.65	0.34	7.53	-3.30	0.30	2.28
80	8.94	0.65	0.35	8.19	-3.74	0.30	2.19
90	9.49	0.66	0.34	8.71	-4.16	0.31	2.09
100	10.00	0.68	0.32	9.07	-4.53	0.36	2.00

Table S4: Electrokinetic data for sample Cu@Ni.[a] in 3 mM glucose

ν (mV/s)	$\nu^{1/2}$	E_{pa} (V)	E_{pc} (V)	i_{pa} (mA)	i_{pc} (mA)	ΔE (V)	$\lvert i_{pa}/i_{pc} \rvert$
10	3.16	0.59	0.36	3.94	-0.29	0.22	13.45
20	4.47	0.61	0.36	5.34	-0.75	0.25	7.16
30	5.48	0.62	0.35	6.45	-1.18	0.27	5.45
40	6.32	0.63	0.35	7.39	-1.61	0.28	4.59
50	7.07	0.65	0.34	8.02	-1.97	0.32	4.07
60	7.75	0.67	0.33	8.73	-2.37	0.33	3.68
70	8.37	0.67	0.33	9.40	-2.74	0.35	3.43
80	8.94	0.69	0.32	10.06	-3.02	0.37	3.33
90	9.49	0.70	0.31	10.63	-3.34	0.38	3.18
100	10.00	0.69	0.31	11.10	-3.62	0.39	3.07

Table S5: Electrokinetic data for sample Ag@Ni.[a] in 1 mM glucose

ν (mV/s)	$\nu^{1/2}$	E_{pa} (V)	E_{pc} (V)	i_{pa} (mA)	i_{pc} (mA)	ΔE (V)	$\lvert i_{pa}/i_{pc} \rvert$
10	3.16	0.54	0.35	2.25	-0.34	0.19	6.60
20	4.47	0.56	0.34	3.19	-0.82	0.22	3.89
30	5.48	0.57	0.34	3.95	-1.27	0.24	3.11
40	6.32	0.59	0.33	4.62	-1.70	0.26	2.72
50	7.07	0.60	0.32	5.24	-2.11	0.27	2.48
60	7.75	0.61	0.32	5.91	-2.59	0.29	2.28
70	8.37	0.62	0.31	6.65	-3.07	0.30	2.17
80	8.94	0.63	0.31	7.24	-3.41	0.32	2.12
90	9.49	0.63	0.31	7.77	-3.79	0.33	2.05
100	10.00	0.64	0.30	8.25	-4.17	0.34	1.98

Table S6: Electrokinetic data for sample Ag@Ni.[a] in 3 mM glucose

ν (mV/s)	$\nu^{1/2}$	E_{pa} (V)	E_{pc} (V)	i_{pa} (mA)	i_{pc} (mA)	ΔE (V)	$\lvert i_{pa}/i_{pc} \rvert$
10	3.16	0.59	0.35	3.49	-0.40	0.25	8.77
20	4.47	0.61	0.34	4.69	-0.84	0.28	5.61
30	5.48	0.63	0.33	5.62	-1.22	0.30	4.59
40	6.32	0.65	0.32	6.62	-1.59	0.33	4.18
50	7.07	0.66	0.31	7.04	-1.93	0.34	3.66
60	7.75	0.66	0.31	7.69	-2.25	0.35	3.42
70	8.37	0.68	0.31	8.29	-2.55	0.37	3.25
80	8.94	0.69	0.30	8.88	-2.77	0.39	3.21
90	9.49	0.70	0.30	9.38	-3.02	0.40	3.10
100	10.00	0.70	0.30	9.83	-3.25	0.40	3.02

[a] The symbol ν is scan rate. E_{pa} and E_{pc} are the anodic and cathodic peak potential. i_{pa} and i_{pc} are the anodic and cathodic peak current. ΔE is the potential difference between anodic and cathodic peaks. $\lvert i_{pa}/i_{pc} \rvert$ is the absolute value of the anodic/cathodic peak current ratio.

S5 Amperometry Results

Amperometry experiments included studying the anodic current response to incremental glucose addition at a fixed potential. A three-electrode system was used to conduct these experiments with Ti as the working electrode, Ag/AgCl as the reference electrode, and Pt as the counter electrode. The fixed potential used for the amperometry experiments were +0.54 V, +0.62V, and +0.62V for the samples Ni, Ag@Ni, and Cu@Ni, respectively. A calibration curve correlating the current response vs. glucose concentration was plotted to get the limit of detection, linear range of response, and sensitivity of the samples in the presence of glucose. In the amperometry experiments, glucose of known concentration stock solution was added at regular intervals of time. For the amperometry experimental plots, current response (mA) is in the y-axis and time (s) lapsed is in x-axis. The amount of 120 mM glucose stock solution added to the 0.1 M NaOH solution is marked in the amperometry plots. After the addition of glucose, the solution was stirred for 40 s followed by 260 s of stabilization period. The current response to a particular glucose concentration was obtained by averaging the current response readings in the last 30 s of the stabilization period and is used in the calibration curve (discussed in the main article). The amperometry experiments data for the samples Ni, Ag@Ni, and Cu@Ni are plotted in Figures S9(a), S9(b), and S9(c), respectively.

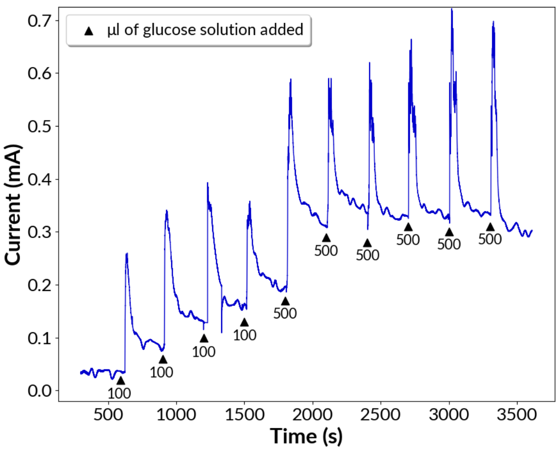

(a)

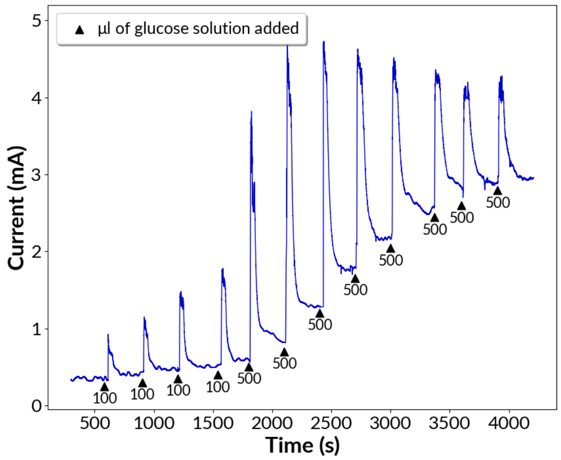

(b)

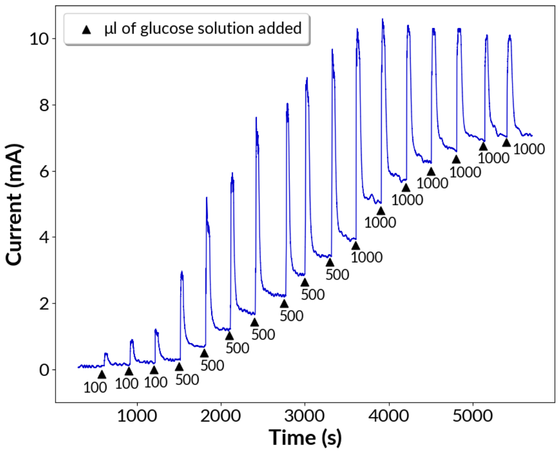

(c)

Figure S9: Amperometric response curves for (a) Ni-coating (b) Ag@Ni-coating and (c) Cu@Ni-coating at the fixed potential of 0.54 mV, 0.62 mV and 0.62 mV, respectively.
